# Ruptured grade 3 mixed ovarian teratoma mimicking acute ovarian torsion: a case report

**DOI:** 10.1093/jscr/rjag468

**Published:** 2026-07-11

**Authors:** Maria Ashafaq, Hamyel Tahir, Bilal Aslam, Dania Hussain, Anas Nasir, Safoora Anjum, Sofia Muryem, Saima Rafique, Imran Nazir, Misbah Aslam, Leka Sree Anbarasan, Fazeela Bibi, Shamsul Qamar, Ahmad Sanan, Said Hamid Sadat

**Affiliations:** University College of Medicine & Dentistry (UCMD), University of Lahore, 1 km Defense Road, Bhoptian Chowk, 54792, Lahore, Pakistan; CMH Medical College, 4 A link Wahdat Road, Lahore, Pakistan; University of Lahore, 1 km Defense Road, Bhoptian Chowk, 54792, Lahore, Pakistan; United Medical and Dental College, House D229, Sector 31/E, Lukhnow Society, Karachi 74900, Pakistan; Community of Medicine, Boys' Hostel, Sheikh Zayed Medical College, Rahim Yar Khan 64200, Pakistan; University of Lahore, 1 km Defense Road, Bhoptian Chowk, 54792, Lahore, Pakistan; Peshawar Medical College, Warsak Rd, Sher Ali Town, Peshawar 25160, Pakistan; University of Lahore, 1 km Defense Road, Bhoptian Chowk, 54792, Lahore, Pakistan; University of Lahore, 1 km Defense Road, Bhoptian Chowk, 54792, Lahore, Pakistan; Al Tibri Medical College, Old Thana Road, Memon Goth, Gadap Town, Malir, Karachi, Pakistan; Indira Gandhi Medical College and Research Institute, Thilaspet, Kathirkamam, Puducherry, 605009, India; Jinnah Medical and Dental College (JMDC), 22-23 Shaheed-e-Millat Road, Karachi 74000, Pakistan; Rural Health Center (RHC), Takhtabad, Union Council Takhtabad, Town-II, Peshawar, Khyber Pakhtunkhwa, 24401, Pakistan; Department of Medicine, Khyber Medical College, Peshawar Pakistan; Department of General Surgery, Kabul University of Medical Sciences, Kabul University Road, Kabul 1001, Afghanistan

**Keywords:** ovarian mixed teratoma, immature teratoma grade 3, ovarian torsion, germ cell tumour, mature teratoma

## Abstract

Ovarian teratomas are common germ cell neoplasms, yet the simultaneous occurrence of mature and high-grade immature components in a single ovary remains rare. We report a 29-year-old female presenting with acute pelvic pain. Imaging suggested ovarian torsion; however, emergency laparotomy revealed a ruptured 130 mm mass. Histopathology confirmed a mixed teratoma with 25% Grade 3 immature neuroectodermal elements (FIGO Stage IC2) and 75% mature elements. Postoperative alpha-fetoprotein was 90.5 ng/ml. Following multidisciplinary review, the patient underwent completion surgery and adjuvant chemotherapy (BEP). This case underscores the clinical imperative for meticulous pathological grading and the necessity of maintaining a high index of oncological suspicion in the management of acute adnexal events.

## Introduction

Ovarian germ cell tumours (OGCTs) account for ~15%–20% of ovarian neoplasms, with mature cystic teratomas (MCTs) being the most common subtype (~95% of ovarian teratomas; incidence 1.2–14.2/100 000 women/year) [1]. MCTs are typically benign and contain well-differentiated tissues from all three germ layers, whereas immature teratomas (ITs) are malignant and composed of embryonic, mainly neuroectodermal tissue [2]. ITs are rare, representing <1% of ovarian teratomas and ~3% of malignant ovarian tumours [3].

These tumors likely arise from a single totipotent germ cell after the first meiotic division [4]. Clinically, rapid adnexal growth may lead to acute events such as torsion or rupture in up to 16% of cases [5]. Capsular rupture (FIGO IC2) is especially concerning in high-grade disease, increasing the risk of peritoneal seeding, chemical peritonitis, and gliomatosis peritonei [6].

Histopathological grading remains central to prognosis, using Norris or WHO criteria to quantify immature neuroepithelium [7]. While contralateral synchronous teratomas may occur, the coexistence of mature and high-grade immature elements within a single ovary (‘mixed teratoma’) is rare [8]. This case of a Grade 3 mixed teratoma presenting as a surgical emergency highlights the importance of meticulous pathological sampling and a multidisciplinary approach.

## Case presentation and investigations

A 29-year-old nulliparous female, with an unremarkable medical and surgical history, presented to the emergency department with an acute-onset, agonizing pain localized to the left lower quadrant. The patient reported a prodromal phase of intermittent pelvic discomfort over the preceding 14 days, which acutely progressed to a constant, high-intensity pain associated with persistent emesis. Upon clinical examination, the patient exhibited significant abdominal distress with localized peritonitis, characterized by rebound tenderness and involuntary guarding in the infra-umbilical region. A point-of-care urine pregnancy test was negative, excluding ectopic gestation.

Ultrasound showed a markedly enlarged left ovary (109 × 64 mm) with echogenic stroma and peripherally displaced follicles, suggesting stromal edema from venous and lymphatic congestion. Despite preserved Doppler flow, moderate complex free fluid and ovarian enlargement raised concern for evolving torsion, prompting emergency laparotomy.

**Figure 1:**
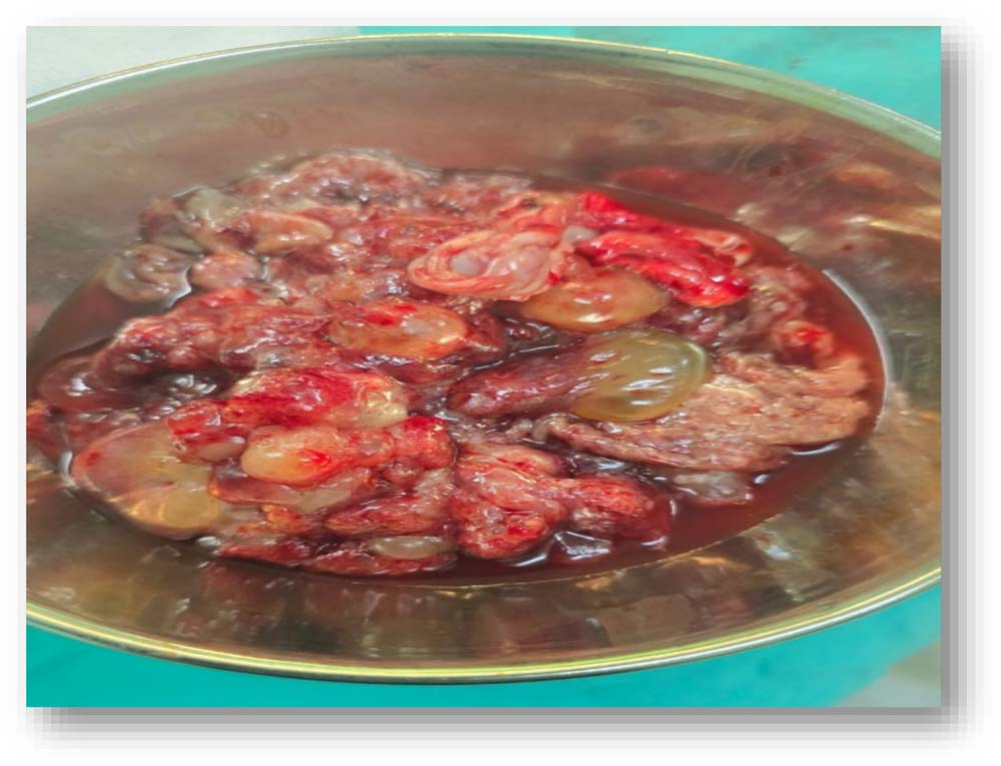
Intraoperative view of the ruptured left ovarian mass.

Intraoperatively, a 130 mm irregular, congested left ovarian mass was identified with heterogeneous, lobulated morphology and multiple cystic areas containing serosanguinous fluid. Capsular rupture with active hemorrhage was noted (Fig. 1). The right adnexa and pelvic peritoneum were unremarkable.

Fertility-sparing left ovarian cystectomy was performed with reconstruction, with preserved viability confirmed by good capillary refill. Ascitic fluid cytology, cyst wall, and omental biopsies were obtained.

The 130 mm tumor showed a mixed germ cell pattern with 75% mature and 25% immature neuroectodermal elements. Primitive neuroepithelial rosettes in >3 low-power fields classified it as a Grade 3 immature teratoma according to Norris criteria.

Staging was pT1c2 (FIGO IC2) due to preoperative capsular rupture. Omental biopsies were negative, and peritoneal cytology showed no malignancy. AFP was elevated (90.5 ng/mL), while β-hCG and LDH were normal.

Following multidisciplinary review, completion left salpingo-oophorectomy with staging laparoscopy was performed. Serosal biopsies were taken, and BEP chemotherapy was initiated.

**Figure 2:**
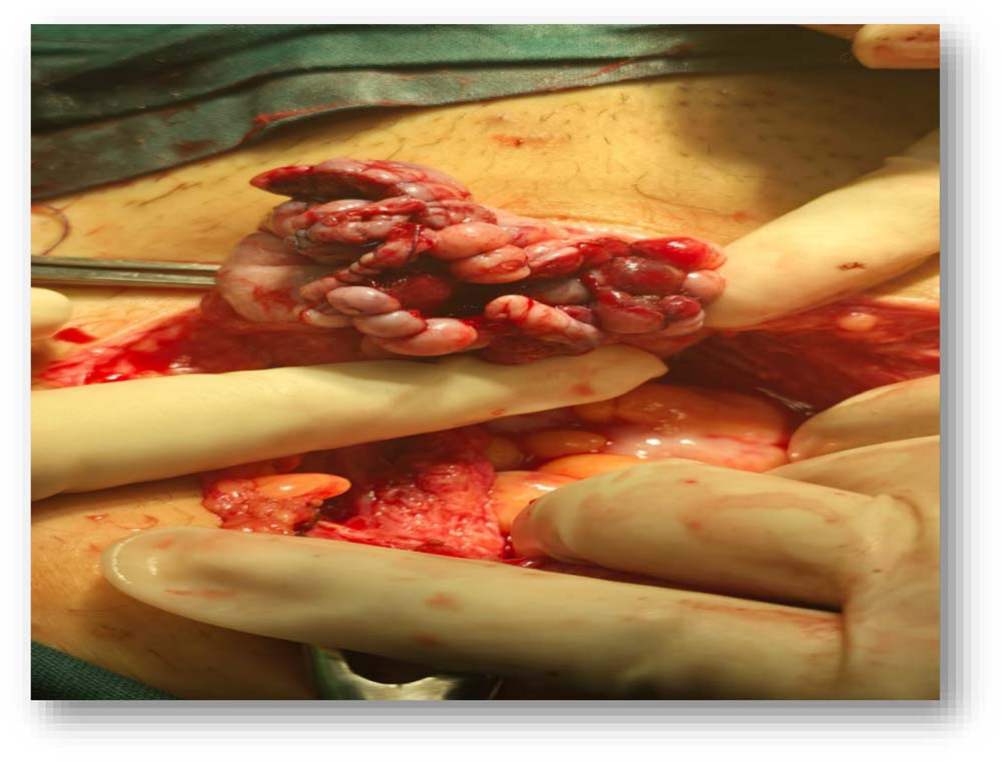
Post-reconstruction appearance of the left ovary.

After three cycles, second-look laparoscopy demonstrated complete response with negative biopsies (Fig. 2). The patient remains in remission under AFP surveillance and imaging follow-up.

## Discussion

The simultaneous manifestation of mature and immature components within an ipsilateral ovary is an infrequently encountered pathological phenomenon. While the malignant transformation of a pre-existing MCT has been suggested by some authors [9], an alternative hypothesis posits a common germ cell origin characterized by asymmetric differentiation [7]. The biological complexity of these mixed lesions is further underscored by the presence of high-grade neuroepithelial elements, a finding that mandates meticulous histological evaluation to establish a definitive diagnosis.

From a diagnostic perspective, the differentiation between teratoma subtypes via preoperative imaging is often obscured by overlapping features. Pathognomonic indicators of MCT, such as macroscopic fat and coarse calcifications, are frequently well-visualized; however, ITs are more typically associated with large, predominantly solid, or heterogeneously cystic architectures [8]. In the present case, the characteristic sonographic and radiological landmarks were effectively masked by the acute capsular rupture and associated hemoperitoneum, a complication that mandated immediate surgical intervention and obscured the underlying malignancy.

Prognosis in malignant germ cell tumours depends on histological grade and stage at presentation [10]. Grade 3 tumours are more aggressive, with a high risk of recurrence without adjuvant therapy, making platinum-based chemotherapy essential even in early-stage disease [11]. Serum AFP serves as a useful biomarker, often reflecting yolk sac components or highly proliferative immature tissue [12].

Fertility-sparing surgery remains standard in young patients [13]; however, Grade 3 tumours with capsular rupture (FIGO IC2) carry a higher risk of peritoneal spread [6, 11]. This highlights the need for strong clinical suspicion, accurate staging, and a multidisciplinary approach to optimize outcomes while preserving fertility [14].

## Conclusion

This case highlights the need for strong oncological suspicion in large adnexal masses, especially with solid components or rupture. Even a small focus of high-grade immature neuroepithelium significantly changes prognosis and thus, necessitates the platinum-based chemotherapy. A multidisciplinary approach is essential for accurate FIGO staging and optimal outcomes. Early recognition helps balance radical surgery with fertility preservation in young patients. Careful pathological sampling and structured follow-up are key to improving survival and reducing recurrence in high-grade OGCTs.

## Data Availability

All data supporting the findings of this case report are included within the manuscript. No additional datasets were generated or analyzed. Clinical information has been anonymized and derived from patient records.
